# PMP22 related neuropathies: Charcot-Marie-Tooth disease type 1A and Hereditary Neuropathy with liability to Pressure Palsies

**DOI:** 10.1186/1750-1172-9-38

**Published:** 2014-03-19

**Authors:** Barbara W van Paassen, Anneke J van der Kooi, Karin Y van Spaendonck-Zwarts, Camiel Verhamme, Frank Baas, Marianne de Visser

**Affiliations:** 1 Department of Clinical Genetics, Academic Medical Center, Meibergdreef 9, 1105 AZ, Amsterdam, the Netherlands; 2 Department of Neurology, Academic Medical Center, Meibergdreef 9, 1105 AZ, Amsterdam, the Netherlands; 3 Department of Genome Analysis, Academic Medical Center, Meibergdreef 9, 1105 AZ, Amsterdam, the Netherlands

**Keywords:** Peripheral myelin protein 22 (PMP22), Charcot-Marie-Tooth disease type 1A (CMT1A), Hereditary Motor and Sensory Neuropathy type Ia (HMSN Ia), Hereditary Neuropathy with liability to Pressure Palsies (HNPP), Demyelinating, Clinical description, Genetic counselling

## Abstract

*PMP22* related neuropathies comprise (1) *PMP22* duplications leading to Charcot-Marie-Tooth disease type 1A (CMT1A), (2) *PMP22* deletions, leading to Hereditary Neuropathy with liability to Pressure Palsies (HNPP), and (3) *PMP22* point mutations, causing both phenotypes. Overall prevalence of CMT is usually reported as 1:2,500, epidemiological studies show that 20-64% of CMT patients carry the *PMP22* duplication. The prevalence of HNPP is not well known. CMT1A usually presents in the first two decades with difficulty walking or running. Distal symmetrical muscle weakness and wasting and sensory loss is present, legs more frequently and more severely affected than arms. HNPP typically leads to episodic, painless, recurrent, focal motor and sensory peripheral neuropathy, preceded by minor compression on the affected nerve. Electrophysiological evaluation is needed to determine whether the polyneuropathy is demyelinating. Sonography of the nerves can be useful. Diagnosis is confirmed by finding respectively a *PMP22* duplication, deletion or point mutation. Differential diagnosis includes other inherited neuropathies, and acquired polyneuropathies. The mode of inheritance is autosomal dominant and *de novo* mutations occur. Offspring of patients have a chance of 50% to inherit the mutation from their affected parent. Prenatal testing is possible; requests for prenatal testing are not common. Treatment is currently symptomatic and may include management by a rehabilitation physician, physiotherapist, occupational therapist and orthopaedic surgeon. Adult CMT1A patients show slow clinical progression of disease, which seems to reflect a process of normal ageing. Life expectancy is normal.

## Introduction

*PMP22* related neuropathies can be divided in three groups. The first group is caused by a *PMP22* duplication, and constitutes the majority of Charcot-Marie-Tooth disease type 1A (CMT1A). The second group is caused by a *PMP22* deletion, leading to Hereditary Neuropathy with liability to Pressure Palsies (HNPP). The third group is composed of neuropathies related to point mutations in the *PMP22* gene and is classified as CMT1A or CMT1E.

## *PMP22* duplication – Charcot-Marie-Tooth disease type 1A (CMT1A)

### Disease name

Charcot-Marie-Tooth disease type 1A (CMT1A). Synonyms: Hereditary Motor and Sensory Neuropathy type Ia (HMSN Ia). Orphanumber ORPHA101081.

### Definition

Charcot-Marie-Tooth disease (CMT), also known as Hereditary Motor and Sensory Neuropathy (HMSN) [1], encompasses a clinically and genetically heterogeneous group of disorders characterized by predominantly distal muscle weakness and atrophy, and sensory loss. The disease was first described in 1886 by Charcot and Marie in France and independently by Tooth in Great Britain and was named after them [2,3]. CMT is the commonest inherited neuromuscular disorder. More than 45 causative genes have been identified [4,5]. Despite the genetic heterogeneity, the clinical phenotype is relatively homogeneous [6]. Classification is based on a combination of neurophysiologic characteristics, inheritance pattern, and underlying genetic cause [7]. Based on neurophysiologic findings three different subtypes are distinguished, i.e. the demyelinating type (CMT1) defined by a motor conduction velocity (MCV) of the median or ulnar nerve of less than 38 m/s, the axonal type (CMT2) with MCV above 38 m/s [8], and an intermediate type [9,10]. The existence of the latter is a controversial topic. Several definitions exist for the term “intermediate”. It has been used in individuals with a MCV between 30 and 40 m/s [10,11], in individuals with clinical and histopathological evidence of both abnormal myelin and axon abnormalities [11,12] and in families in which members have either a demyelinating phenotype or an axonal phenotype, like families with X-linked CMT due to GJB1-mutations, with male members showing a demyelinating type and female an axonal type [9,13]. CMT can be inherited in an autosomal dominant, autosomal recessive and X-linked manner. Previously, histopathological features were also applied to distinguish demyelinating from axonal neuropathy [1,14]. However, nowadays, a nerve biopsy for diagnosis is considered to be obsolete. Further subclassification is based on the underlying genetic cause. CMT1A is the most common subtype of CMT1. This autosomal dominantly inherited demyelinating form of CMT is caused by a 1.5 Mb duplication on chromosome 17p11.2 [15,16], containing the gene coding for peripheral myelin protein 22 (PMP22) and thus leading to three copies of the *PMP22* gene [17-20].

### Epidemiology

Overall prevalence of CMT is usually reported as 1:2,500 [21]. Several more recent epidemiological studies [22-25] reported prevalences of CMT ranging from 1:1,214 (in Norway) to 1:6,500 (in the United Kingdom). Epidemiological studies show that 19.6% to 64.7% of CMT patients carry the *PMP22* duplication [22,25], which gives calculated prevalences of CMT1A in the range of 1:3,800 to 1:12,500.

### Clinical description

The age of onset of CMT1A is mainly in the first two decades [26,27] and most frequently in the first 10 years of life [26-30]. Exceptions occur as onset at birth as a floppy infant [28] or with congenital foot deformities [26,31]. Late onset at 76 years [27] is mentioned and a case report describing siblings diagnosed at age 69 and 65 years [32]. CMT1A usually presents as the “classical CMT” phenotype [7,26]. The typical presenting symptom is difficulty walking or running, due to weakness of the distal leg muscles [26,28,33]. CMT patients usually have distal symmetrical muscle weakness and wasting, legs (“stork appearance”) more frequently and more severely affected than arms. Calf hypertrophy instead of atrophy may also be found [26,28,29]. Proximal weakness can be observed in up to 28% in knee extensors [27-29,34,35]. Foot deformity, usually pes cavus with hammertoes, is a cardinal feature in CMT1A patients, with up to 90% of patients presenting with pes cavus [29]. In about one third of patients this can be the presenting symptom [26]. Clawing of the hands may occur, but is usually milder. Skeletal deformity of the spine (scoliosis) is reported in 4-35% of patients [26-29]. Upper extremity symptoms include muscle weakness, primarily of intrinsic muscles, and atrophy. Decreased manual dexterity was found to be a common finding among subjects with CMT1A [36]. Sensory symptoms are usually less prominent and can be subtle [8,26,37,38]. Impaired sensation is usually found in a stocking-glove distribution with the legs more frequently and more severely affected than the arms. Large fibre sensory involvement leads to proprioceptive loss, causing balance difficulties [37]. Small fibre sensory loss can be present with complaints of cold feet and decreased temperature discrimination sense [37]. Pain in CMT patients is more common than previously recognized, with 55-70% of CMT1A patients reporting pain [39,40], which is considered predominantly nociceptive. Severe fatigue is reported in 60% of adult patients [41] and is also reported in children (24%, in contrast to 14% of general school-based population) [42]. Tremor, especially of the hands, can be a feature [26-29,37]. In some cases the tremor can be quite severe. Several CMT1A patients with tremor are described as having a Roussy-Lévy phenotype [26,43,44]. However, the original Roussy-Lévy family carried a mutation in the MPZ gene [45]. Nonprogressive sensorineural hearing impairment is described in CMT1A patients [46]. Sixty-one percent of the paediatric CMT1 patients (16/18 with *PMP22* duplication) showed significantly impaired speech perception ability, although they showed normal or near-normal sound detection [47]. Vestibular impairment seems to be frequent in patients with CMT [48]. Phrenic nerve involvement, causing dyspnoea when lying flat or nocturnal hypoventilation, is infrequent in CMT1A, but has been reported in some, severely affected, patients (in 5% [26] and 3.5% [28], respectively). CMT may predispose to obstructive sleep apnoea [49]. Only rarely patients have normal reflexes, mostly they are absent (in up to 46-75% of patients) or depressed [26-29]. Hypertrophy of nerves is a feature of CMT1A, rarely clinically present and mostly established by pathology [44] or imaging studies [50-53].

There is large clinical variability between patients, even within the same family [54,55]. Overall, 1-7% of patients become wheelchair dependent [27,28,35]. A walking aid (cane, crutches or walker) is needed by 3-14% of patients [27,28,35,38]. Dejerine-Sottas syndrome (in the past also known as Hereditary Motor and Sensory Neuropathy type III) is a hereditary neuropathy with early onset and severe presentation. Currently it is not considered a different entity, but part of the phenotypic spectrum of CMT1, because the underlying genetic defects are known CMT1 genes, like the *PMP22* duplication [56,57]. There are case reports on severely affected CMT patients demonstrating the presence of two mutations in two different CMT-related genes (“double trouble”) [58,59]. On the other side of the spectrum, patients can be asymptomatic (1.6% to 17% [26-28]) or only exhibit mild symptoms, like foot deformity, leading to no or only mild problems with walking [26,27]. However the term asymptomatic is questionable, as asymptomatic individuals might not experience complaints but clinical examination may show pes cavus and/or ankle jerk areflexia [27].

Although always considered a disease affecting the peripheral nervous system, a recent report on 15 CMT1A patients described central nervous system involvement [60]. Decreased white matter volume was found in 73%, with minimal, predominantly executive, cognitive disorders in 77%.

Electrophysiologically, CMT1A is characterized by a homogeneous and diffuse motor and sensory nerve conduction slowing [6,27,38,54,61,62]. Due to a combined effect of dysmyelination and axonal dysfunction and loss, sensory nerve action potentials (SNAP) are also frequently reduced in the arms and reduced to absent in the legs [27,29,38].

Nerve biopsies show decreased density of myelinated nerve fibres, most pronounced in biopsies taken in the first year of life. The mean g-ratio (axon diameter versus fibre diameter) is significantly lower than normal [63]. Characteristic onion bulb formation occurs after the age of six [44,63-65]. Axonal loss is also found in childhood [65]. Abnormal myelination probably extends over the whole nerve length. The most severe pathological changes were seen distally in nerves, but there were also changes in the proximal nerves and in the roots [66].

An overview of the key features of CMT1A is presented in Table 1.

**Table 1:** Key features of CMT1A and HNPP

	**CMT1A**	**HNPP**
	**Duplication of PMP22**	**Deletion of PMP22**
**Clinical features**	Age of onset mainly in first two decades	Painless attacks of numbness, muscular weakness, and atrophy, recurrent and focal
	Presenting symptom is difficulty walking or running	
		Preceded by minor compression on nerve
	Distal symmetrical muscle weakness and wasting, legs > arms	
		Age at onset mostly in the second or third decade
	Pes cavus very frequent	Pes cavus found in 4-47% of patients
	Sensory symptoms (stocking-glove distribution) usually less prominent, legs > arms	
		Full recovery in 50% of episodes, usually in days to weeks
	Pain more common than previously recognized	Sequelae rarely severe
		Large intrafamilial clinical variability
	Reflexes absent or depressed	
	Large clinical variability between patients, even within family	
**Electrophysiological features**	Homogeneous and diffuse MCV and SCV slowing	Increase in distal motor latencies, especially of median and peroneal nerve
	CMAP amplitudes reduced, especially distally in the legs	
		Focal motor slowing at entrapment sites
	SNAP amplitudes frequently reduced to absent	
		MCV normal to slightly reduced in other segments
		SCV decreased and SNAP amplitudes often reduced
**Neuropathological features**	Abnormal myelination over the whole nerve length	Segmental de-and remyelination
	Onion bulbs	Tomacula pathologic hallmark, but not pathognomonic
	Decreased density of myelinated nerve fibres	
		Variable large-fibre loss

CMAP = compound muscle action potential. MCV = motor conduction velocity. SCV = sensory conduction velocity. SNAP = sensory nerve action potentials.

### Aetiology

CMT1A is predominantly caused by a 1.5 Mb duplication on chromosome 17p11.2 [15,16] that includes the *PMP22* gene [17-20]. Cases of CMT1A with different sized duplications, most including the *PMP22* gene [67,68] or a copy number variant upstream of *PMP22* are reported [69]. Occasionally (<5%) point mutations in the gene are found (see “*PMP22* point mutations”). Mutations in the *PMP22* gene had previously been identified as the Trembler mouse mutation. Identification of an identical mutation in a CMT family showed that the *PMP22* gene is also responsible for CMT1A [70].

Gene dosage of *PMP22* is the proposed mechanism, supported by the finding that increased PMP22 protein [71] and elevated *PMP22* messenger RNA (mRNA) was found in CMT1A patients in sural nerve biopsies [72]. The *PMP22* gene encodes a 22-kD protein that comprises 2 to 5% of peripheral nervous system myelin. PMP22 is produced primarily by Schwann cells. It is expressed in the compact portion of essentially all myelinated fibres in the peripheral nervous system [73]. The exact function of PMP22 is still not elucidated. Studies in injured nerve suggested a role during Schwann cell growth and differentiation [73].

CMT1A has been considered a primary demyelinating neuropathy, as also shown by low conduction velocities on electrophysiology in CMT1A patients. However, it has become clear that axonal dysfunction determines clinical disease severity [35,38,74,75]. Onion bulbs seen on morphological studies of nerve biopsies are a sign of de- and remyelination. Evidence is accumulating that CMT1A might be a disorder of dysmyelination instead of demyelination, meaning myelination is delayed and normal myelination is never reached [76-79].

### Diagnosis (diagnostic criteria and algorithms) and diagnostic methods

If a patient presents with a chronic motor and sensory polyneuropathy, CMT should be one of the differential diagnoses. In the assessment of any patient with a polyneuropathy, the first issue to consider is whether the polyneuropathy is hereditary, by taking a detailed family history about polyneuropathies, walking difficulties or pes cavus. The mode of inheritance is autosomal dominant, but one should be aware of asymptomatic or non-diagnosed family members and a *de novo *rate of 10% [80,81].

Electrophysiologic evaluation is needed to determine whether the polyneuropathy is demyelinating, axonal or intermediate. Sonography of median nerves can be helpful. It was shown that median nerve cross-sectional area (CSA) was significantly increased in CMT1A compared to CMT2 [50,52,53], and CSA correlated with nerve conduction slowing in CMT1A [52].

In autosomal dominantly inherited uniformly demyelinating sensorimotor polyneuropathy, the *PMP22* duplication, the most common cause of CMT1, is tested first. In severe cases “double trouble” (the presence of two mutations in two different CMT-related genes) can be present. Currently, with the knowledge and easy accessibility of DNA diagnostics, nerve biopsy is obsolete.

### Differential diagnosis

**Figure 1:**
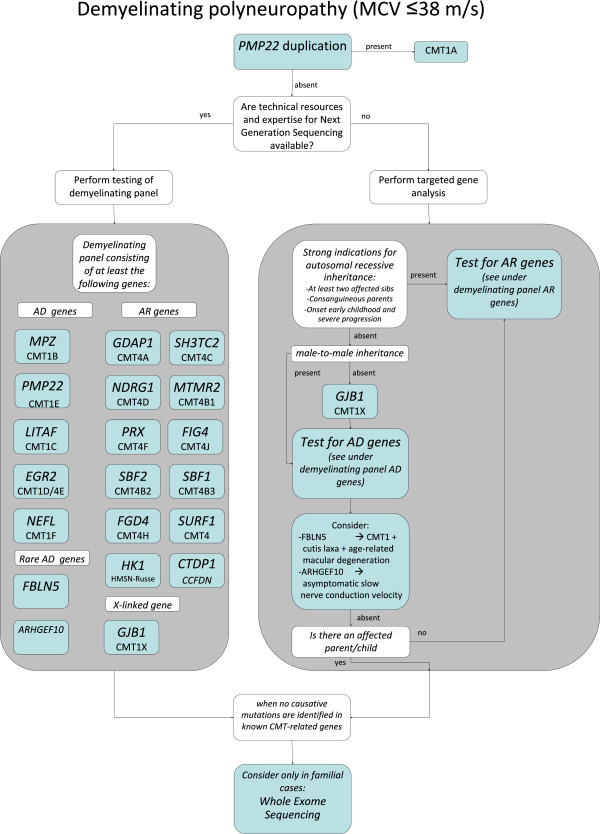
**Algorithm for genetic testing of patients with demyelinating neuropathy.** Analysis should always start with testing for *PMP22* duplication. If negative, a panel containing genes associated with CMT1 should be tested if technical recourses and expertise for Next Generation Sequencing (NGS) is available, otherwise targeted gene analysis as depicted on the right is the next step. When no pathogenic mutation is identified, Whole Exome Sequencing should be considered in familial cases only.

Also in a patient without a positive autosomal dominant family history presenting with a uniformly demyelinating sensorimotor polyneuropathy, CMT1A should be considered and tested first. If DNA-testing for the *PMP22* duplication is negative, other forms of CMT1 should be considered. An algorithm for genetic testing of patients with demyelinating polyneuropathy is presented in Figure 1. In view of the large number of genes involved in CMT, a gene by gene analysis is time consuming. With the introduction of Next Generation Sequencing (NGS) in clinical diagnostics the development of NGS panels for CMT is expected [82-84]. The first diagnostic step in patients with demyelinating polyneuropathy should remain testing for *PMP22* duplication. If negative, all the genes should be sequenced in one run (panel). Whole exome sequencing (WES) is not recommended as a first screen since the costs are still high and the sensitivity of this technology is not yet high enough. WES should only be applied to those familial cases where screening of the affected family members has proven negative. The results of the NGS panel should be discussed with geneticists with expertise in the CMT-area. Key issue in the analysis is the likelihood of the sequence variant to be pathogenic and to correspond with the patient’s phenotype and inheritance pattern. This incorporates cosegregation analysis in affected family members. If technical resources or expertise for NGS is not available, a candidate gene approach is also presented in Figure 1.

Demyelinating polyneuropathy can also be a sign of autosomal recessive metachromatic leucodystrophy [85], Refsum’s disease [86], Krabbe’s disease [87], X-linked adrenomyeloneuropathy [88], Pelizaeus-Merzbacher syndrome [89] and Lowe syndrome [90], but in addition other characteristic symptoms and signs will be present. Acquired causes associated with segmental demyelinating neuropathy, e.g. diabetes mellitus, and chronic inflammatory demyelinating polyneuropathy (CIDP) should also be considered. Typically, CIDP shows a subacute or fluctuating course, multi-focal demyelinating features on electrophysiology, high protein levels in cerebrospinal fluid, no pes cavus and a negative family history [91,92]. However, in clinical practice, symptoms of CIDP and CMT can overlap [93]. Establishing the diagnosis is important, since CIDP is treatable. Diabetes mellitus is the most common cause of neuropathy [94] and should always be considered in a polyneuropathy patient. A distal symmetrical sensory polyneuropathy is the most common pattern of diabetic neuropathy [95]. Diabetic neuropathy is usually axonal, but can also show demyelinating features on electrophysiology [91]. As opposed to CMT, diabetic neuropathy has predominantly sensory and autonomic manifestations [94].

### Genetic counselling and antenatal diagnosis

CMT1A is inherited in an autosomal dominant fashion. Thus offspring of CMT1A patients have a chance of 50% to inherit the *PMP22* duplication from their affected parent. Anticipation is described in several case studies [96-98]. However, in our opinion this rather reflects phenotypic variability within a family, which is a well known phenomenon in CMT1A [54,55]. The variability is likely, at least in part, due to genetic modifiers (see section “unresolved questions”).

When the diagnosis of an inherited condition is made in a family, issues like family planning and testing of relatives at risk can arise. The best way of handling these issues, is nondirective counselling. This is based on the autonomy of the patient to make the decision that serves his/her best interests, after receiving nondirective information about benefits and disadvantages of testing, considering for instance family planning, work related choices and insurance issues. If clinically unaffected family members request predictive testing and are found to carry the *PMP22* duplication, it is likely that they will develop some features, since the penetrance of the disease is nearly 100% [99]. There is consensus about not testing minors at risk of an inherited disorder without treatment or preventive options, because of the chance of psychological harm to the child [100,101]. Prenatal testing on foetal DNA is possible. During pregnancy foetal DNA can be obtained by chorionic villus sampling or by amniocentesis, both with a risk of miscarriage due to the sampling. Requests for prenatal testing for conditions that do not affect life span or intellect are not common. Requests for prenatal testing should be managed in a multidisciplinary team, involving genetic counsellors, psychologists and (paediatric) neurologists [102]. Pre-implantation Genetic Diagnosis (PGD) is a technique used to identify genetic defects in embryos created through in vitro fertilization before pregnancy. PGD is sparsely undertaken for CMT1A [103]. In some countries it is not considered an indication for PGD [104].

### Management including treatment

Treatment consists of supportive care and may include management by a rehabilitation physician, physiotherapist, occupational therapist and orthopaedic surgeon.

Supportive care for the legs includes exercise training, shoe inlays, orthopaedic shoes and orthoses. In case of severe skeletal deformities surgical correction may be considered. However, evidence is limited; a Cochrane review concluded that methodologically sound trials are required for any of these physical interventions [105,106]. Orthopaedic surgery to correct severe pes cavus or hammertoes may be helpful [107-109]. Likewise, robust evidence is not present. Symptomatic treatment for the arms has not been studied extensively. Wearing a thumb opposition splint may improve manual dexterity in CMT [110]. Tendon transfer surgery is moving a tendon from its original attachment to a new one to restore the action of the transferred muscle and improve function. It may improve thumb opposition of patients with CMT [111,112]. Symptomatic drug treatment for positive sensory symptoms and for muscle cramps may be useful, but has not been investigated specifically for CMT.

Co-existence of diabetes mellitus in a CMT1A patient is described to exacerbate symptoms of the peripheral neuropathy [113,114], and therefore optimal control of blood sugar should be strived for in a CMT patient with diabetes. Toxin or medication-induced worsening of pre-existing peripheral neuropathy is a generally known phenomenon [115]. The use of neurotoxic agents, especially vincristine, can have a devastating effect, even in low dose [116,117] and should be avoided in patients with CMT.

Perspectives on future therapeutic developments are discussed in the “unresolved questions” section.

### Prognosis

As mentioned, axonal dysfunction determines clinical disease severity [35,38,74,75]. Nerve conduction velocities are found to remain stable in children after the age of six years [35,77,118,119]. There are only a few natural history studies done in adult CMT1A patients [35,118,120], all showing slow clinical progression. Only one 5-year natural history study included age-matched controls [35] and showed that baseline strength and compound muscle action potential (CMAP) amplitudes were lower in patients than in controls, while adult CMT1A patients and controls had a similar decline of strength and of CMAP amplitudes over time, suggesting that progression in patients may reflect a process of normal ageing. Physical disability however increased over time in the patient group and not in the control group. This may be explained by fewer reserves in patients, as baseline strength and CMAP amplitudes were lower. Also skeletal deformations may progress. Studies about the natural history in children are ongoing. Life expectancy is normal.

### Unresolved questions

Why an increase or decrease in *PMP22* gene copy number results in CMT1A or HNPP is still unknown. PMP22, also known as growth arrested specific gene 3 (GAS3), is a transmembrane protein and its expression increases upon growth arrest [121] and in proliferating cells the levels are very low. The highest expression of *PMP22* is detected in the Schwann cells of compact myelin. It is possible, that in these cells *PMP22* serves another function. There is no unifying hypothesis how a *PMP22* copy number variation or mutation leads to disease. In case of point mutations a toxic effect of misfolded protein is suggested, but this does not hold for the duplication and deletion of a normal copy of *PMP22*. In that case one can speculate that *PMP22* interacts with other myelin components and stoichiometry is essential for proper function. However, there is no experimental data for this hypothesis.

The variability of the CMT1A phenotype, even within families [27], suggests the presence of modifiers - genetic and/or external factors - but these have not been identified thus far. We expect that part of the variation of the phenotype is due to genetic factors. Severely affected “double trouble” cases (the presence of two mutations in two different CMT-related genes) are reported [58,59] and show that known CMT genes can act as modifiers. Systematic screening of CMT genes in large cohorts of patients is necessary to identify the more common variants that affect the phenotype. With targeted NGS or whole exome sequencing [122] probably becoming the standard in DNA diagnostics in the near future, it is expected that variants in CMT related genes will be identified, which, together with a pathogenic mutation, determine the phenotypic expression of CMT in an individual patient. Thus far, the number of reports on these double trouble cases is low. We recommend that DNA screening of other CMT genes is performed in severely affected patients carrying a *PMP22* duplication.

Variation in pathways involved in the immune system and its effect on nerve degeneration or regeneration may also affect the severity of the CMT phenotype. Analysis of transgenic models for CMT have excluded a role for B- and T-cells in the *PMP22* overexpressing mice [123]. However, alterations in the innate immune system can affect the severity of the neuropathy. Heterozygous deletion of monocyte chemoattractant protein-1 (MCP-1) does affect axonal properties [124] and it was shown that inhibition of the complement system has a major effect on nerve degeneration and regeneration [125,126].

Therapy for CMT1A thus far consists of supportive care only. Since the CMT1A phenotype is shown to result from a gene dosage effect [17-19], it is hypothesized that regulating the *PMP22* gene dosage may be a therapeutic target. Two compounds that have been found to regulate *PMP22* mRNA levels in rodent models are progesterone [127,128] and ascorbic acid [129]. Ascorbic acid did not show a beneficial effect in several randomised controlled trials [130-134]. Onapristone, a progesterone receptor antagonist, reduced *PMP22* mRNA levels and improved the phenotype in rodents [127]. However, clinical trials were not initiated due to the known serious side effects in humans [135]. No reports about progesterone antagonist treatment in CMT have been published since.

A pilot study undertaken by Sahenk et al. [136] showed that neurotrophin-3 (NT-3) improved the phenotype of the Trembler-J mouse and also led to slight improvement of sensory and reflex scores in CMT1A patients. Sahenk and colleagues proposed NT-3 treatment via adeno-associated virus (AAV) delivery to muscle and they showed in the Trembler-J mouse model that rAAV1.NT-3 therapy resulted in measurable NT-3 secretion levels in blood and improvement in motor function, histopathology, and electrophysiology of the peripheral nerves [137].

Cell line assays for high-throughput screens are a valuable new tool to select candidates from large numbers of existing compounds. Regulatory elements in the *PMP22* gene are found that direct expression of *PMP22*[138,139]. An intronic regulatory element was coupled to the luciferase gene, creating a high-throughput screen platform [140]. A multitude of compounds were tested on this assay, resulting in the identification of four compounds that decreased *PMP22* mRNA and protein [140]. Follow-up assays followed by animal trials are the next step.

Two transgenic rodent models, the C3-PMP mice and the CMT-rat closely resemble human CMT1A and may be appropriate models for therapeutic studies [79,141].

An important issue in developing treatment with drugs that showed benefit in animal studies is when to proceed to trials in patients. It can be argued that positive results in animal studies should be replicated first, before clinical trials in patients are developed [142].

Effective drug treatment may be directed at normalization of the myelination process, although improvement of the axonal function should be the ultimate goal. In view of this, therapies modulating the innate immune response should not be neglected. Since axonal degeneration already starts in childhood, drug treatment beginning early in life is expected to be most beneficial.

## *PMP22* deletion - Hereditary Neuropathy with liability to Pressure Palsies (HNPP)

### Disease name

Hereditary Neuropathy with liability to Pressure Palsies (HNPP). Synonymes: tomaculous neuropathy. Polyneuropathy, familial recurrent. Orphanumber ORPHA640.

### Definition

A deletion of the 1.5 Mb region on chromosome 17p11.2, the same region that is duplicated in CMT1A [143] causes the autosomal dominantly inherited disorder Hereditary Neuropathy with liability to Pressure Palsies (HNPP). HNPP is an episodic, multifocal neuropathy. The typical clinical presentation is that of recurrent transient pressure palsies without pain, but with focal motor and sensory symptoms in the territory of a single nerve or the brachial plexus [144].

### Epidemiology

The prevalence of HNPP is not well known [37]. Prevalences of 7.3 per 100,000 [23] to 16 per 100,000 [145] are reported.

### Clinical description

HNPP typically leads to episodic, painless, recurrent, focal motor and sensory peripheral neuropathy [33]. It may cause attacks of numbness, muscular weakness, and atrophy [146]. Many episodes are preceded by minor compression on the affected nerve [146], for instance prolonged positioning of the limb. The most vulnerable nerves are the peroneal and ulnar nerves (30-48% and 21-28%, respectively), followed by the brachial plexus (12-27%), radial nerve (4-13%) and median nerve (4-11%) [144,147,148].

Age at onset of first HNPP symptoms is mostly in the second or third decade, with a large range from birth (although only two cases are described, one with an transient Erb’s paresis [145] and one with neuropathy of the peroneal nerve and pes cavus [149]) to the eighth decade [144,145,148,150-153]. Most patients (60-70%) present with a single, focal, acute neuropathy [144,148,150]. Cranial nerves are affected rarely [154]. Transient palsies of the facial [155-157], trigeminal [155,158], hypoglossal [155,159] and recurrent nerve [160] have been described. Although not being a typical transient nerve palsy, sensorineural hearing impairment of postnatal onset with progression beyond presbyacusis has been reported [46]. Other uncommon presentations include recurrent short-term positional sensory symptoms, progressive sensorimotor mononeuropathy of the peroneal nerve, chronic sensory polyneuropathy, chronic sensorimotor polyneuropathy and subacute peripheral quadriparetic episodes (initial diagnosis was chronic idiopathic demyelinating polyneuropathy) [144]. Also a Davidenkow phenotype (scapuloperoneal syndrome) is described [161].

On clinical examination, besides muscle weakness, atrophy and/or sensory signs in the affected nerves, reduced or absent tendon reflexes, mostly the ankle jerks, can be noted [144,154]. Pes cavus may be found in 4-47% [144,148,150-152,157]. The symptoms from acute neuropathy usually disappear in days to weeks [146]. Full recovery occurs in 50% of episodes. Remaining symptoms are rarely severe [162]. Chronic motor deficits after nerve palsies are noted in 10% to 15% [144,148,150]. Other chronic symptoms like cramps, paresthesias and exercise-induced myalgia have been described [150].

There is large clinical variability between HNPP patients. At the mild end of the spectrum, patients can be clinically asymptomatic. In different studies the percentages of asymptomatic family members of index patients varied from 6% to 23% [144,148,150,152,153]. At the severe end of the spectrum patients have residual symptoms after nerve palsies, which can mimic a CMT phenotype [146,163].

Electrophysiological studies show a characteristic pattern [144,147,164-167]. Essentially all HNPP patients, whether symptomatic or not [164] show an increase in distal motor latencies, especially of the median and peroneal nerves [166]. Most patients show focal motor slowing at entrapment sites, more commonly in the ulnar nerve at the elbow than in the peroneal nerve at the fibular head [144,147,164,166]. In other segments, MCV is normal in most cases, but can be slightly reduced in some. Sensory nerve conduction velocities are often decreased and sensory nerve action potential amplitudes are often reduced [166,167].

Nerve biopsy shows segmental de- and remyelination and varying large-fibre loss [168]. Tomacula, which are massive redundancies or overfolding of layers of variable thickness in the myelin sheath, are the pathologic hallmark [33], but are not pathognomonic.

Central nervous system white matter lesions have been reported in isolated patients and in several members of a large family with HNPP [169-171]. A recent report on 15 HNPP patients described central nervous system involvement [60]. Decreased white matter volume was found in 71%, with minimal, predominantly executive, cognitive disorders in 64%.

An overview of the key features of HNPP is presented in Table 1.

### Aetiology

A 1.5 Mb deletion on chromosome 17p11.2 [143,154] is found in approximately 85-90% of patients with clinical evidence of HNPP. Most of the CMT1A- or HNPP-associated rearrangements in 17p11.2 are recurrent and mediated by nonallelic homologous recombination, although rare cases of HNPP have deletions of different size but always including the *PMP22* gene [68]. HNPP is an autosomal dominant disease. The *de novo* rate is probably 20% [164]. Rare cases have mutations in the *PMP22* gene. These mutations often result in stop codons and thus give rise to a null allele [172].

The gene dosage hypothesis is supported by the finding of decreased PMP22 protein [71] and decreased *PMP22* mRNA expression levels in HNPP patients [173] that correlated with phenotype [174]. It is hypothesized that the focal symptoms of HNPP are caused by reversible conduction block (defined by >50% reduction of CMAP amplitudes between distal and proximal sites of stimulation) [152]. Structural abnormalities at the node of Ranvier explain the changes in axonal excitability, and these abnormalities would predispose the nerves to conduction block when subjected to pressure or stretch [175]. In a mouse model heterozygous for *Pmp22*, conduction block was more rapidly induced by mechanical compression on the nerve in comparison to normal nerves [176] and recovery was slower. They found focal axonal constrictions within tomacula. Reduced axonal diameter raises resistance to action potential propagation and thus predisposes these axons to conduction block. When the nerve is compressed, it may cause even further thinning of axons.

### Diagnosis (diagnostic criteria and algorithms) and diagnostic methods

Guidelines for diagnosis of HNPP are proposed by Dubourg et al. [154]. A clinical manifestation of acute, painless, recurrent peripheral nerve palsies is typical for HNPP. However, also with more uncommon presentations (see “clinical description”) HNPP should be considered. A family history consistent with autosomal dominant inheritance is often present, but is not a prerequisite considering the *de novo* rate of 20% and asymptomatic occurrence of the disease. Electrophysiology of HNPP patients helps in establishing the diagnosis. Sonography of nerves show nerve hypertrophy at entrapment sites [177,178], although not always significant [52].

If HNPP is suspected, DNA testing for the deletion of the *PMP22* gene, followed by sequencing of the *PMP22* gene if no deletion is found, can confirm the diagnosis. Like in CMT1A, nerve biopsy is essentially obsolete.

### Differential diagnosis

Pressure palsies are commonly the result of acquired physical compression of peripheral nerves, mostly of the median nerve at the wrist (carpal tunnel syndrome), the peroneal nerve at the fibular head and the ulnar nerve at the elbow [162]. Screening for HNPP in 50 patients with isolated carpal tunnel syndrome showed that none of the patients had the *PMP22* deletion [179]. The suspicion of HNPP should be higher if a patient suffers from more than one episode of compression neuropathy, if there is also an unexplained polyneuropathy present and if there is a family history of neuropathy [162]. Mononeuropathies may also be caused by compression due to a tumour, bleeding or abscess. In case of a neuropathy of the brachial plexus with a positive family history hereditary neuralgic amyotrophy (HNA) is considered most commonly [146,163] and can be associated with mutations in the SEPT9 gene [180]. The nerve palsy in HNPP is painless, whereas the brachial plexopathy in HNA is preceded by severe pain. In case of a negative family history the sporadic form of HNA, idiopathic neuralgic amyotrophy, should be considered [163].

### Genetic counselling and antenatal diagnosis

HNPP is an autosomal dominantly inherited disorder. For general aspects see corresponding paragraphs in the CMT1A section. In our experience, predictive testing, prenatal testing and PGD are not commonly requested.

### Management including treatment

Treatment is currently symptomatic. Management during a pressure palsy may include transient bracing. If a pressure palsy is not transient but residual, the bracing may need to be permanent. Patients should be informed about avoiding activities that are risk factors for pressure palsies. These activities include prolonged sitting with legs crossed, occupations requiring repetitive movements of the wrist, prolonged leaning on elbows and rapid weight loss [162,181]. Toxin or medication-induced worsening of pre-existing peripheral neuropathy is a generally known phenomenon [115]. Vincristine, used in chemotherapy, has been reported to exacerbate HNPP [182]. No systematic controlled study of surgical decompression of nerves has been done. Given the vulnerability of the peripheral nerves in patients with HNPP, surgery is generally considered unfavourable [146].

Perspectives on future therapeutic developments are discussed in the "unresolved questions" section.

### Prognosis

No natural history studies of HNPP exist. The prognosis is therefore unsure. In a study addressing age associated changes in electrophysiology [152], a reduction in CMAP with increasing age at examination was observed in nerves vulnerable to entrapment. This nerve-specific CMAP reduction likely results from a history of repetitive focal compression of the nerve. It is however not mentioned how the CMAP reduction correlates with symptoms. Recovery from acute neuropathy is often complete, although it can take months and if chronic symptoms persist, they are usually mild. Residual symptoms after nerve palsies can resemble a CMT phenotype [146,163]. Symptomatic individuals have the frustration and disability associated with recurrent pressure palsies [162]. Life expectancy is normal.

### Unresolved questions

As for CMT1A, the phenotype is highly variable in HNPP, suggesting the presence of modifiers - genetic and/or external factors. So far none are identified.

Treatment is still only symptomatic. To our knowledge no trials have been undertaken. As HNPP is caused by a decreased dosage of *PMP22*, increasing the gene dosage may be a therapeutic target, as decreasing the gene dosage is for CMT1A [146]. Stimulation of the endogenous *PMP22* by enhancing the regulatory elements of *PMP22* might be one option. Another option is to introduce another copy of the *PMP22* gene into the peripheral nerve by gene therapy. An obstacle is that increasing the dosage of *PMP22* above a certain level will cause CMT1A.

## *PMP22* point mutations

Point mutations in *PMP22* are found in a minority of patients suspected to have CMT or HNPP and the phenotype may vary from mild HNPP to severe CMT1 [183,184]. Clinical overlap between CMT1 and HNPP is also described, for instance in patients with the frameshift mutation Gly94fsX222 (c.281_282insG) [148,185]. For all general issues considering CMT1A and HNPP, see previous sections.

According to some classifications CMT caused by *PMP22* mutations is also called CMT1A [7], while others consider this a separate disease entity called CMT1E [99,184,186]. One could argue to classify CMT caused by *PMP22* point mutations as a separate entity, because the genetic defect is different from CMT caused by *PMP22* duplication. An extra argument for considering this a separate disease entity is that *PMP22* point mutations can phenotypically lead to CMT as well as HNPP or even an overlap between the two phenotypes. In this perspective, HNPP due to *PMP22* point mutations should also be seen as a separate entity from HNPP due to *PMP22* deletion.

Several types of *PMP22* point mutations are described (missense, nonsense, frameshift and splice-site mutations [5]). Controversy about the pathogenicity of the Thr118Met mutation exists. It is reported as a benign polymorphism, as an autosomal recessive inherited mutation or as an autosomal dominant mutation, with HNPP as phenotype [187]. Very rare autosomal recessive neuropathy (CMT4), caused by homozygosity for point mutations in *PMP22*[188,189] has been reported.

## Conclusions

*PMP22* related neuropathies are the most prevalent amongst the inherited neuropathies. Knowledge about clinical presentation of CMT1A and HNPP and the expanding possibilities of genetic testing is a prerequisite for all neurologists and geneticists dealing with inherited neuropathy patients. Research on therapy has so far largely focussed on CMT1A, because of its prevalence and the rationale of decreasing the gene dosage of *PMP22* which could potentially reverse the phenotype. One should keep in mind that a *PMP22* directed therapy can not be applied to all cases of CMT. Therefore the genetic subtyping of CMT is essential to select those cases that might be eligible for targeted treatment strategies, as soon as these treatments become available.

## Abbreviations

AD: Autosomal dominant; AR: Autosomal recessive; ARHGEF10: Rho guanine nucleotide exchange factor 10; CIDP: Chronic inflammatory demyelinating polyneuropathy; CMAP: Compound muscle action potential; CMT: Charcot-Marie-Tooth disease; CMT1: Charcot-Marie-Tooth disease type 1; CMT1A: Charcot-Marie-Tooth disease type 1A; CSA: Cross-sectional area; CTDP1: C-terminal domain of RNA polymerase II subunit A, phosphatase, subunit 1; DNA: Deoxyribonucleic acid; EGR2: Early growth response 2; FBLN5: Fibulin 5; FGD4: FYVE, RhoGEF and PH domain-containing protein 4; FIG4: FIG4 homolog of S. cerevisiae; GDAP1: Ganglioside-induced differentiation-associated protein 1; GJB1: Gap junction protein, beta 1; HK1: Hexokinase 1; HMSN Ia: Hereditary motor and sensory neuropathy type Ia; HMSN: Hereditary motor and sensory neuropathy; HNA: Hereditary neuralgic amyotrophy; HNPP: Hereditary neuropathy with liability to pressure palsies; LITAF: Lipopolysaccharide-induced tumor necrosis factor-alpha factor; Mb: Mega base pairs; MCV: Motor conduction velocity; MPZ: Myelin protein zero; MTMR2: Myotubularin-related protein 2; NDRG1: N-myc downstream-regulated gene 1; NEFL: Neurofilament protein, light polypeptide; NGS: Next generation sequencing; PGD: Pre-implantation genetic diagnosis; PMP22: Peripheral myelin protein 22; PRX: Periaxin; RNA: Ribonucleic acid; SBF1: SET-binding factor 1; SBF2: SET-binding factor 2; SH3TC2: SH3 domain and tetratricopeptide repeat domain 2; SNAP: Sensory nerve action potential; SURF1: Surfeit 1; WES: Whole exome sequencing.

## Competing interests

The authors declare that they have no competing interests.

## Authors’ contributions

BWvP: drafting of the manuscript. AJvdK: revising of the manuscript. KvS-Z: revising of the manuscript. CV: drafting the parts on electrophysiology and revising of the manuscript. FB: revising of the manuscript. MdV: initiator and revising of the manuscript. All authors read and approved the final manuscript.
